# Animal-Morphing Bio-Inspired Mechatronic Systems: Research Framework in Robot Design to Enhance Interplanetary Exploration on the Moon

**DOI:** 10.3390/biomimetics9110693

**Published:** 2024-11-13

**Authors:** José Cornejo, Cecilia E. García Cena, José Baca

**Affiliations:** 1 Escuela Técnica Superior de Ingeniería y Diseño Industrial, Universidad Politécnica de Madrid, Ronda de Valencia, 3, 28012 Madrid, Spain; cecilia.garcia@upm.es (C.E.G.C.); jose.baca@tamucc.edu (J.B.); 2 Centre for Automation and Robotics (UPM-CSIC), Ronda de Valencia, 3, 28012 Madrid, Spain; 3 Department of Engineering, College of Engineering and Computer Science, Texas A&M University-Corpus Christi, Corpus Christi, TX 78414, USA

**Keywords:** aerospace robotics, bio-inspired systems, space exploration, ISRU, moon

## Abstract

Over the past 50 years, the space race has potentially grown due to the development of sophisticated mechatronic systems. One of the most important is the bio-inspired mobile-planetary robots, actually for which there is no reported one that currently works physically on the Moon. Nonetheless, significant progress has been made to design biomimetic systems based on animal morphology adapted to sand (granular material) to test them in analog planetary environments, such as regolith simulants. Biomimetics and bio-inspired attributes contribute significantly to advancements across various industries by incorporating features from biological organisms, including autonomy, intelligence, adaptability, energy efficiency, self-repair, robustness, lightweight construction, and digging capabilities-all crucial for space systems. This study includes a scoping review, as of July 2024, focused on the design of animal-inspired robotic hardware for planetary exploration, supported by a bibliometric analysis of 482 papers indexed in Scopus. It also involves the classification and comparison of limbed and limbless animal-inspired robotic systems adapted for movement in soil and sand (locomotion methods such as grabbing-pushing, wriggling, undulating, and rolling) where the most published robots are inspired by worms, moles, snakes, lizards, crabs, and spiders. As a result of this research, this work presents a pioneering methodology for designing bio-inspired robots, justifying the application of biological morphologies for subsurface or surface lunar exploration. By highlighting the technical features of actuators, sensors, and mechanisms, this approach demonstrates the potential for advancing space robotics, by designing biomechatronic systems that mimic animal characteristics.

## 1. Introduction

The Acceleration, Reconnection, Turbulence and Electrodynamics of the Moon’s Interaction with the Sun (ARTEMIS) mission proposed by NASA aims to explore the Moon and beyond. As outlined in the strategic plan [1,2], there are two important goals: (1) expand human presence on the Moon for sustainable long-term exploration and development; and (2) investigate the lunar surface. These could provide chances to extract lunar resources, resulting in safer and more efficient operations that are less reliant on supplies from Earth. As a result, NASA has projects related to in-situ resource utilization (ISRU) through partnerships with industry and academia [3]. Extraction and mining initiatives enhance the ability to find and exploit lunar rock resources from the regolith. Moreover, the development of chemical and thermal processes may provide options for breaking down natural minerals and compounds found on the Moon and turning them into items for human consumption [4]. Therefore, NASA will begin establishing the ARTEMIS Base Camp [5] on the lunar surface, allowing for long-term stays and unsupervised scientific activities [6]; it also serves as a valuable platform for technical advancement. Other mission-enhancing capabilities include sophisticated solar and fission power systems [7] and even automated manufacturing technologies, like 3D printing, using regolith-based construction materials [8].

The ARTEMIS mission is focused on the lunar south pole, an area with more extreme environments than any other location humans have encountered. The landscape is heavily cratered, with portions drenched in sunlight and others in darkness. The craters are frigid, but the elevated parts can become exceedingly heated. These characteristics increase risk while providing bigger scientific rewards. Experiments will yield fresh insights about the nature and genesis of polar volatiles [9]. The south pole region is reported to have reserves of water ice that future explorers could utilize for drinking, cooling, oxygen production, and rocket fuel [10]. The advancement of ISRU technologies enables longer-duration human missions to distant destinations [11], which can begin with a pilot plant based on the Volatiles Investigating Polar Exploration Rover (VIPER) [12] and the Polar Resources Ice Mining Experiment (PRIME-1) [13] missions. These missions could assist process selection and operations [14], aiming to validate the fundamental capabilities and subsystems for producing water and/or oxygen from indigenous materials [15].

Therefore, the Lunar and Planetary Institute (LPI) has identified two specific objectives for ISRU operations [16]: (1) To track environmental changes caused by human activities(sensors will be installed at various distances from an outpost or settlement). Lunar exploration provides a valuable chance to assess the effects of human presence on biological and organic contamination and to develop mitigation strategies [17]. By creating techniques to test and reduce contamination, this activity will aid in the preparation of human missions to Mars [18]; (2) To examine and understand each phase of the impact process. The following fundamental discoveries are crucial to this interpretation: (i) the distribution of primary ejecta from craters and basins [19]; (ii) the geochemical deconvolution of the megaregolith to determine the bulk composition of the crust [20]; and (iii) the utilization of the basin, larger crater ejecta, and central peak compositions to deduce crustal stratigraphy [21]. Moreover, intact lunar craters can serve as a natural laboratory for studying the impact process across a wide variety of sizes [22]. There are three types of soils on the lunar surface [7], lunar mare, highlands, and mixing zones [8], which can be reproduced as a simulant using low-Ti mare basalt and high Ca highland anorthosites [9]. These are under manufactured to test the capabilities of robots in extracting resources [10] such as oxygen, iron, titanium, or chromium (structural elements), and helium (fuel) [11].

Deep space exploration, extravehicular activities, and the expansion of space science research depend on the development of planetary robots [23]. There is ample evidence that the use of these technologies minimizes the expense and danger associated with space activities. A growing number of countries have declared that their next phase of space missions will involve exploring the Moon and Mars. Thus, robots in space must overcome huge challenges caused by extreme temperatures, radiation, microgravity, and ultrahigh vacuum [24]. In addition, using an intelligent robot to explore extraterrestrial regolith (ER) offers several benefits and may be the most important path to enable long-term survival. An emerging trend in ER exploration will be human–robot cooperation, in which robots perform tasks that are challenging for humans to accomplish, whereas robots handle simpler or riskier ones. When astronauts arrive at celestial bodies, this trend will become a powerful reinforcement [25]. According to Tao Zhang et al. [26], the planetary regolith sampler (PRS) is a kind of equipment that is typically mounted on a lander or rover during planetary exploration [27]. It is thought to expand the range of applications for planetary robots. The PRS can penetrate, gather, transport, and store regolith samples. The design and development of such multifunctional devices have caught the interest of scientists and engineers worldwide due to their enormous potential for application in deep space exploration. However, it is difficult to establish a fully functional PRS due to the enormous environmental changes among celestial bodies [28].

Finally, for decades, engineers have employed biomimetics and bio-inspiration to improve products and procedures across many industries [29,30]. Thus, several features shared by biological organisms, such as autonomy and intelligence, response-stimuli adaptability, energy efficiency, ability to self-repair, robustness, lightweight construction, and digging tasks could be essential for space systems. Those features fit into the “Biomimetics” research field explored by the Advanced Concepts Team [31] of the European Space Agency (ESA) [32], and also by Innovative Advanced Concepts led by NASA [33]. In extraterrestrial environments, many challenges and irregular surface textures pose significant issues and limitations for traditional wheeled robotic vehicles [34]. Therefore, this paper presents a framework based on a wide range of bionic technology concepts, each with unique features based on particular specifications. Therefore, the main contribution of this manuscript lies in the proposal of foundations for the design and development of bio-inspired planetary space robots for applications on the Moon [35,36]; in addition, it depicts a literature review oriented to the sand locomotion of animals that can be replicated to design biomimetic systems, classified by subsurface and surface exploration tasks. We have identified two types of mechatronic systems, that can be reproduced based on animals [37] and adapted to sand environments: (1) limbed robots bio-inspired on crabs, moles, lizards, and spiders; and (2) limbless robots bio-inspired by snakes and sandworms. Biomimetics has demonstrated its capacity to offer numerous beneficial insights and enhance traditional space systems by introducing innovative and creative principles, even when facing space’s extremely harsh environmental conditions [38].

The manuscript is structured as follows: Section 2 describes the materials and methods used for data search. Section 3 shows the selected animal species according to their morphological adaptations, followed by an analysis of robotic systems that mimic anatomical and locomotion features of the animals mentioned above, resulting in a novel design methodology. Section 4 discusses the advantages of bio-inspired robotics for lunar applications, focusing on (i) mechanics and materials science and (ii) mechatronics and control systems. The paper concludes with a summary of findings and future works. 

## 2. Materials and Methods

**Figure 1:**
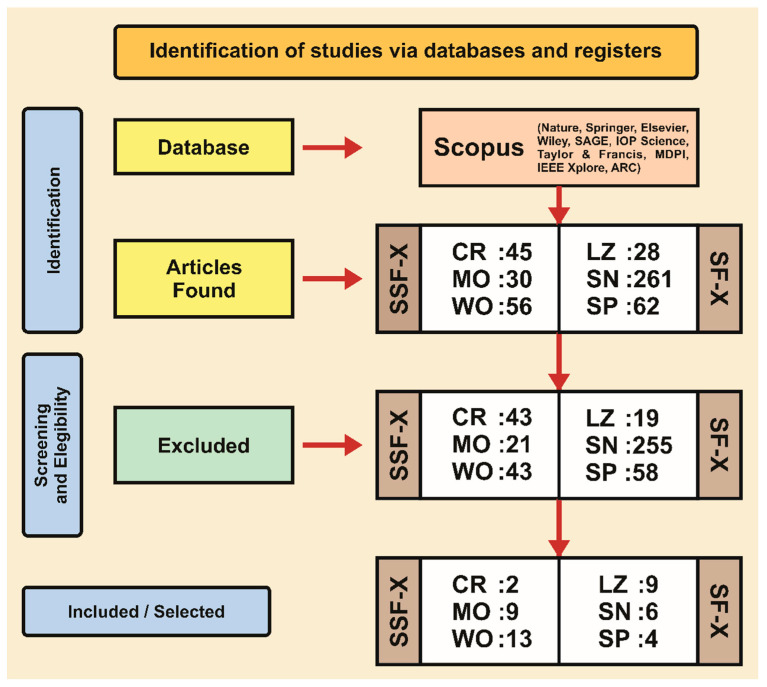
Adapted PRISMA flow diagram of the search process. CR: Crab, MO: Mole, WO: Worm, LZ: Lizard, SN: Snake. SP: Spider, SF-X: Surface exploration, SSF-X: Subsurface exploration. The numbers mean the quantity of published articles.

This section presents the research methodology (Figure 1), which has been inspired by the trending topic in robotics that is related to biomimetics. Therefore, the Kitchenham guidelines [39] were applied in the formulation process of this literature review to classify the published articles about underground sand-living animal morphologies (described in Section 3) that can be replicated as a bio-inspired systems. The search was designed according to the Population, Intervention, Comparison, Outcomes, and Context (PICOC) criteria [40] and adapted for bio-inspired systems (Table 1).

This study examined scientific contributions published up to July 2024. EndNote was used to store and manage references. To begin, the most appropriate literature databases (DBs) were chosen to increase the chances of discovering highly relevant articles. This database includes Scopus (www.scopus.com, accessed on 1 July 2024) because it covers the most important publishers, such as Nature (www.nature.com, accessed on 3 July 2024), Springer (www.springer.com, accessed on 5 July 2024), Elsevier (www.elsevier.com, accessed on 7 July 2024), Wiley (www.wiley.com, accessed on 8 July 2024), SAGE (www.sagepub.com, accessed on 11 July 2024), IOP Science (www.iopscience.iop.org, accessed on 13 July 2024), Science (www.science.org, accessed on 14 July 2024), Taylor & Francis (www.tandfonline.com, accessed on 16 July 2024), MDPI (www.mdpi.com, accessed on 17 July 2024), IEEE Xplore (ieeexplore.ieee.org, accessed on 19 July 2024), and Aerospace Research Central (arc.aiaa.org, accessed on 20 July 2024). So, Table A1—Appendix A depicts the search protocol’s structure for the paper’s identification stage, which is divided into 2 blocks and 5 parts. 

In the screening and eligibility stage, articles published in journals, books, research reports, and conference proceedings from the aforementioned DB were included. Additionally, at the beginning, the articles were examined by reading the title and abstract. The following eligibility criteria are shown in Table 2 as follows:

Additionally, the anatomy and physiology features related to the biomechanics of sand-animals’ musculoskeletal systems during locomotion activities [41] are described, such as (a) grabbing-pushing: the teeth destroy the substrate, while the forelimbs compact it to both sides, thus enabling dig caves [42], (b) wriggling: it can produce stable reverse force in the non-linear and unconstructed environment, and the animal does not be affected by the substrate pressure [43], (c) undulating: the body undergoes large-amplitude axial fluctuations and uses the fluid characteristics of the substrate to produce propulsion [44], and (d) rolling: the animal moves along a surface by revolving or turning over and over to perform a fast motion [45].

Consequently, the locomotion-gait and engineering design of each biomimetic robot has been described and classified as species of subsurface and surface exploration (see Table 3). These systems cover a wide area of land, expanding the regions explored by humans. While exploring, they are capable of collecting many different types of data, such as geological composition, atmospheric measurements, terrain mapping, etc. A space planetary robot mainly covers these features [46,47], such as (i) can move forward in the soil, although the body is buried, (ii) lightweight, and (iii) enough autonomy to explore by itself. 

## 3. Results

**Figure 2:**
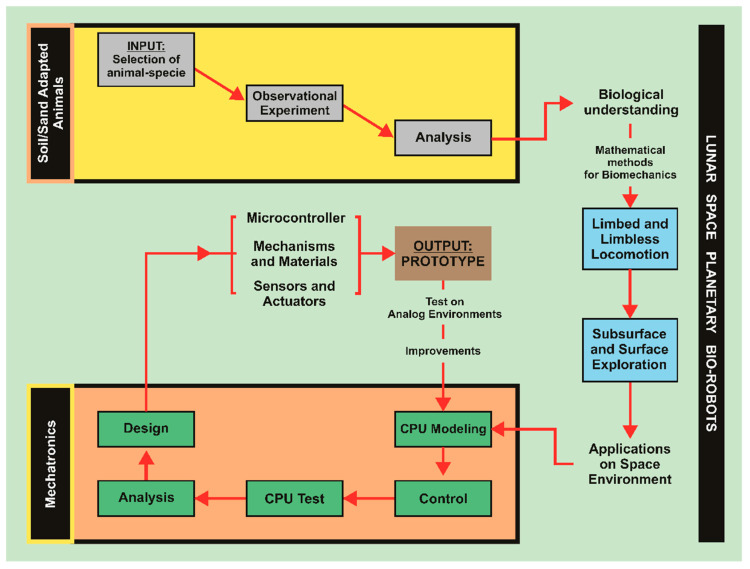
Novel proposal of design methodology for space planetary bio-robots, it starts with the INPUT: Selection of animal-specie, and finishes with the OUTPUT: Prototype. Note: Analog Environment is defined as terrestrial locations that exhibit geological or environmental conditions analogous to celestial bodies, like the Moon or Mars. Source: Original contribution.

In summary, for decades, engineers have employed biomimetics and bio-inspiration to improve products and procedures across a wide range of industries. Thus, several features shared by biological organisms, such as autonomy and intelligence, response-stimuli adaptability, energy efficiency, ability to self-repair, robustness, lightweight construction, and digging tasks, are essential for space systems. Many challenges and irregular surface textures are common in extraterrestrial environments, which cause problems and constraints for traditional wheeled robotic vehicles [34]. Hence, a wide range of bionic technology concepts exist, each with unique qualities based on particular specifications. Based on a thorough analysis of the literature presented in Section 2, we can state a proposal to standardize the methodology (following the VDI 2221 [91] and VDI 6220 [92] guidelines) for designing robots that are able to perform exploration activities on the subsurface and surface of the Moon (based on ISO 49.140—Space systems and operations [93], AIAA-92-1515—Standards for space automation and robotics [94], and AIAA S-066-1995—Standard vocabulary for space automation and robotics [95]), ISO/TC 299—Robotics [96], and ISO 18458:2015—Biomimetics: Terminology, concepts and methodology [97]. As depicted in Figure 2, the concept starts with the observation of the soil/sand-adapted animal behavior, and then, it can be replicated using mathematics to explain the biomechanics. After that, the limbed or limbless option must be selected, and then, a computer (CPU) model combined with control techniques can be created until the design of the prototype is achieved, taking into account mechanisms, materials, sensors, and actuators, managed by microcontrollers. 

This section shows a technical summary of the included/selected documents after the search methodology was performed in July 2024 (Figure 1). It started with the identification stage according to the use of research protocol (Table A1—Appendix A) in Scopus Database, and then the results were screened using the analysis criteria mentioned in Table 2. 

### 3.1. Subsurface Exploration

It requires specialized systems capable of penetrating the regolith and navigating underground tunnels and lava tubes [98]. Bio-inspired burrowing robots are particularly well-suited for this task, reaching depths and providing access to subsurface ice deposits and geological formations [99,100]. Equipped with temperature and moisture sensors, these robots can map subsurface ice deposits with accuracy [101]. Navigating underground environments is critical for studying lunar lava tubes, and potential habitats for future lunar bases. Robots inspired by burrowing animals can traverse these tubes, providing real-time data on their structure and stability [102]. They can navigate tunnels with small diameters and cover long distances per deployment, offering invaluable data for assessing the feasibility of using lava tubes as protective shelters [103].

#### 3.1.1. Crab

Starting with the search methodology (Table A2—Appendix B), in the identification stage, 45 matching articles were found in the Scopus database. Subsequently, in the screening stage, 43 articles were excluded as they did not meet the study analysis criteria (Table 2), leading to 2 articles (Figure 1 and Table A3—Appendix C). Summarizing the information contained in the selected articles, the robot designs report 2-leg pair systems, each of them is driven by a slider-crank linkage with a freely rotating linear bearing, and then, the resulting trajectories of each leg pair create a cycle of insertion, a rapid sweeping or rotation of the central rod, and subsequent retraction. Thus, two robots are described.

The design of Robot 1 covers each leg pair linkage, which is driven by a single brushed DC gearmotor. Besides, a cuticle component of the robot was designed to emulate the function of the arthrodial membrane. These cuticles are made of latex rubber sheeting and clamped between two rigid layers on both the baseplate and bearing housing. Also, the four triangular legs are stretched and retracted in a plane throughout the legs’ sweeping trajectory [48]. In addition, the prototype of Robot 2 with vanes and uropods is presented. The burrowing vanes are actuated by internal E381 radio control servos. Metalized anti-static film was tested and found to have an appropriate combination of flexibility and toughness. The anti-static film used in this project is 0.079 mm thick, consisting of a 0.013 mm polyester layer and a 0.066-mm layer of polyethylene [49].

After studying bio-inspired robotics based on “Crabs” that perform subsurface exploration, we select a particular specie called Pacific Mole Crab (*Emerita analoga*) [104], which is able to develop these steps for underground locomotion as follows: using anterior legs and rear-facing uropods, can quickly burrow backward, (tail first), into the soil, excavating the substrate as it digs underground [105]. The limbs are organized into two main groups. Group 1 consists of the uropods and second leg pair, and Group 2 being pairs 3–5. Group 1 moves with a counterclockwise power stroke, excavating material above the body followed by a clockwise recovery stroke with the appendages folded in Group 2 rotates in the reverse direction propelling the body forward with a clockwise power stroke followed by a clockwise recovery stroke and excavates material below the body. Furthermore, Group 1 operates at twice the frequency of Group 2, completing twice the amount of strokes in low depths [106].

#### 3.1.2. Mole 

Starting with the search methodology (Table A2—Appendix B), in the identification stage, 30 matching articles were found in the Scopus database. Subsequently, in the screening stage, 21 articles were excluded as they did not meet the study analysis criteria (Table 2), leading to 9 articles (Figure 1 and Table A3—Appendix C). Summarizing the information presented in the selected articles, the robot designs report two types of systems based on forelimbs and mandibles that perform burrowing tasks, which permits efficient digging locomotion. Thus, six robots are described.

Applications of two mechanisms are presented based on forelimbs: Robot 1 presents a design of a single-DOF forelimb system called cable-driven burrowing force amplification mechanism (BFAM) based on Stephenson’s six-link mechanism, which connects the claw toe to the steering engine [50]. Also, Robot 2 works with a digging sequence structured as follows: (1) pull the front body, (2) impact force, (3) excavation, and (4) gather both paws [51].

Additionally, there are mole’s mandible-based designs. Robot 3 shows a single-drive linkage mechanism related to the occlusal pattern and skeletal-muscular structure of mole incisors that expand, contact, and bite [52]. Robot 4 presents an excavation system, which is a combined drill bit and forelimbs structure [53,54]. The body section is composed of three parts in total, a fixed body, a rotating body, and a shaft [55]. The shaft and the rotating body rotate simultaneously when the motor operates. The fixed body and the rotating body have screw patterns that mesh with each other like bolts and nuts [56]. Besides, Robot 5 performs the excavation sequence structured as follows: (1) blade expansion and drill bit forward, (2) excavation, (3) blade folding and drill bit reverse, (4) forelimb forward-moving and spread, (5) forelimb backward and debris removal [57]. Consequentially, Robot 6 presents a shaft and rotating body that are simultaneously rotated by the motor. The rotating body moves up and down by the screw pattern of the fixed body [58].

After studying bio-inspired robotics based on “Moles” that perform subsurface exploration, we select a particular specie called Grant’s Golden Mole (*Eremitalpa granti*) [107], which is able to develop these steps for underground locomotion as follows: digging with front claws, pushing sand backward under abdomen, which is retracted forward and upward. Then, the head and shoulders press forward and upward, sustained by front legs trailing occasionally used to promote forward motion [108].

#### 3.1.3. Worm

Starting with the search methodology (Table A2—Appendix B), in the identification stage, 56 matching articles were found in the Scopus database. Subsequently, in the screening stage, 43 articles were excluded as they did not meet the study analysis criteria (Table 2), leading to 13 articles (Figure 1 and Table A3—Appendix C). Summarizing the information contained in the selected articles, the robot designs show a peristaltic locomotion gait pattern, which starts with (1) anchoring the robot tail, (2) elongating the middle segment, (3) anchoring the head while releasing the tail, (4) releasing the middle segment, and then return to Step 1; the cycle ends with all segments released. Thus, seven robots are described.

Also, it is observed that there are autonomous driller-like systems with flexible-rigid morphologies. Robot 1 [59] is composed of an excavation unit with two universal joints, enabling it to perform curved excavation. The goal is to excavate boreholes for placing environmental sensors, and collecting samples from a particular layer [60,61]. Additionally, it is made up of a propulsion unit (it has pneumatic actuators) [62], and a discharging unit (the soil in the back of the robot is discharged out of the borehole using small DC motors giya-domo-ta RS—/775 gm Series Round Shaft) [63,64]. Additionally, Robot 2 is made of rigid material (316 stainless steel). It consists of five units: the drill unit, front support anchor unit, steering unit, propulsion unit, and rear support anchor unit, and all sections are hydraulically driven. Also, it can achieve two degrees of freedom of steering motion through the extension or contraction, working in the coordination of the four cylinders [65].

In addition, there are systems made of soft materials. Robot 3 presents a Kirigami structure, which pops up when an actuator is radially expanded, forming bristle-like spikes that are perpendicular to the surface and folds back down when deflated, forming a smoother skin structure. It exhibits a greater maximum drag force (improved from 2.1 ± 0.3 N to 5.5 ± 0.5 N in 25 mm hole diameter condition), greater forward displacement, and higher traction (e.g., with a 40 g payload, the 3.7 ± 2.8 cm improved to a 12.5 ± 0.1 cm in six gait cycles). To generate peristaltic motion, the robot needs at least three actuated segments, two radially expanding actuators at each end for anchoring, and one longitudinally expanding actuator at the center for elongation along the direction of locomotion. The contractile actuators (thickness 3 mm) are built in the same way as the extensile actuator without a Kevlar thread. The contractile actuators can both extend longitudinally and radially, depending on the pressure around the body. The three segments are connected to each other by press-fit rigid rings, and very flexible Tygon tubes are routed inside the actuators. A conic shape 3D printed rigid nose to reduce the resistant force in the direction of locomotion. The earthworm robot is actuated pneumatically by using the air from a stationary source [66].

Robot 4 has an origami-shape, constructed by connecting identical cells in series; each cell is an origami ball (with axial and radial deformations). Hence, a linkage structure is designed to provide push and pull forces to actuate the origami ball. Under servomotor actuation, the linkage is able to deform axially from 78 mm to 5 mm and simultaneously deform radially from 0 mm to 85.5 mm. Regarding the material, a polyethylene terephthalate (PETE) film (0.05 mm in thickness) is employed for fabrication because it is an inexpensive, safe, non-toxic, strong, lightweight, and flexible that is 100% recyclable [67]. Likewise, Robot 5 has perceptive artificial skins composed of stretchable electric circuits, which can enable the development of pressure and strain sensors made of deformable micro-channels filled with conductive liquid metal eutectic alloys (EGaIn5 and Galinstan6). It is composed of two pneumatically driven radial soft actuators, located at the front and back (extremes) of the robot (made of silicone materials), and a pneumatically driven central axial actuator. This structural configuration enables the linear extension, or contraction, of the actuator by varying its internal air pressure while preventing, to a significant extent, deformations along the radial dimension. A radial actuator replicates the main features of earthworms’ circular muscles. Also, the shape enables the radial expansion, or contraction, of the actuator by varying its internal air pressure while preventing, to some extent, deformations along the axial dimension. Regarding the fabrication methods for both types of actuators, 3D-printed acrylonitrile butadiene styrene (ABS) molds were used, silicone elastomer (Ecoflex 00-50, Smooth-On), butadiene rubber o-rings, sheets of fiberglass, and pneumatic components. Its length is 130 mm and 35 mm in diameter, with an approximately constant wall-thickness of all the components of 2 mm [68].

Furthermore, Robot 6 demonstrates two active configurations from a neutral state by switching the input source between positive and negative pressure. The peristaltic-soft actuator (PSA) generates a longitudinal force for axial penetration and a radial force for anchorage, through bidirectional deformation of the central bellows-like structure, which demonstrates its versatility and ease of control [70]. The central actuator elongates with positive pressure, causing the elastomeric skin to stretch longitudinally and produce radial compression by pushing the encapsulated fluid inward. Once the pressure has been released, the actuator returns to its neutral position and maintains the shape predefined by the elastomeric skin. To achieve full radial expansion, negative pressure is applied, which compresses the actuator along the longitudinal axis and pushes the fluid radially outward [69]. Robot 7 presents similar features [71].

After studying bio-inspired robotics based on “Sandworms” that perform surface exploration, we select a particular species called Bobbit worm (Eunice aphroditois) [109], which is able to develop these steps for above-ground locomotion. It moves using circular and longitudinal muscles, as well as bristles called chaetae. It can push the chaetae out of its body to grab the sand around it. To move forward, the animal uses its chaetae to anchor the front of its body and contracts the longitudinal muscles to shorten its body, which measures up to 3 m (1500 segments), gradually tapering towards the pygidium [110].

### 3.2. Surface Exploration

It requires essential tasks for detailed mapping of the lunar surface. Equipped with advanced LIDAR and hyperspectral imaging systems [111], these systems can generate high-resolution topographical maps with optimized accuracies, identifying potential landing sites and areas of scientific interest [112]. Covering areas up to 10 km^2^ per day they could enhance the scope and speed of lunar reconnaissance missions [47,113]. Bio-inspired robots can excavate and transport lunar regolith to processing units, converting it into essential resources like oxygen, water, and building materials [114,115], with operational efficiency allowing continuous excavation cycles over a 24-h period [116].

#### 3.2.1. Lizard

Starting with the search methodology (Table A2—Appendix B), in the identification stage, 28 matching articles were found in the Scopus database. Subsequently, in the screening stage, 19 articles were excluded as they did not meet the study analysis criteria (Table 2), leading to 9 articles (Figure 1 and Table A3—Appendix C). Summarizing the information contained in the selected articles, the robot designs report a non- and 4-legged systems that performs a waving locomotion (tripod gait), which permits that the body-weight distribution will affect how much a traveling wave contributes to thrust [117]. Thus, four robots are described.

Robot 1 presents a bio-inspired lizard-vertebral, which is composed of six motors. This allows angular excursions in the body plane and is connected via identical links [72]. The design employs six servomotors and a passive segment (the head), being a total of seven segments [73,74]. Regarding the used material that covers the system, the best option was a two-layer encasement consisting of an outer Lycra spandex sleeve with a single seam enclosing an inner, thin latex sleeve that fit tautly [75,76]. 

In addition, it is observed that there are 4-legged systems, where Robot 2 has soft-amphibious features, and it has 4.0 mm diameter thin and soft McKibben actuators that are light, small, and suitable for a simple system. It uses a sprawling posture to support its body. The upper limbs are typically held horizontally, while the lower limbs are vertical. The combination of multi-actuators bending motion of the leg and body through the crawl gait provides flexible locomotion to produce forward or backward motion [77]. Robot 3 describes a similar mechanical configuration related to Robot 2, but this is a rigid-bodied system composed of 10 servomotors [78]. Finally, Robot 4 has multi-degrees of freedom that covers 24 servomotors [79,80].

After studying bio-inspired robotics based on “Lizards” that perform surface exploration, we select a particular species called the Sandfish Lizard (*Scincus scincus*) [118], which is able to develop these steps for above-ground locomotion: it generates thrust to overcome drag by propagating an undulatory traveling wave down the body [119]. It uses the limbs in a paddling motion as a propulsion motion where the body and tail follow the high-amplitude sinusoidal curve adopted by the head. Its anatomy revealed 26 vertebrae in the trunk and 13 anterior caudal vertebrae in the tail [120]. 

In addition, we can mention that another interesting quadruped-reptile animal that has similar leg-pattern motion is the Turtle, but it is slower than Lizards; thus, its morphology covers a larger supporting polygon that greatly improves its motion stability. Regarding the bionic design process, the Turtle is represented in a robotic system using a multistage topology optimization process. The resulting soft leg structure attained an ideal balance between bending flexibility and standing stiffness; moreover, the proposed leg demonstrated superior performance compared to rigid-link legs with motors at the rotational joints. The flexible beams within the soft leg structure effectively redistributed the load throughout the leg, thereby providing enhanced protection to the servomotor against concentrated forces [121]. The other robot shows a reconfigurable structure designed to seamlessly transition between wheeled, legged, and paddle swimming locomotion modes. This system accomplishes three distinct modes of movement: wheeled locomotion on land, limb-crawling on land, and paddle swimming in water. These capabilities are achieved through composite locomotion strategies, including wheel-arm integration, leg-arm transitioning, broad paddling on the wide side, and precise arm shifts [122]. 

#### 3.2.2. Snake

Starting with the search methodology (Table A2—Appendix B), in the identification stage, 261 matching articles were found in the Scopus database. Subsequently, in the screening stage, 255 articles were excluded as they did not meet the study analysis criteria (Table 2), leading to 6 articles (Figure 1 and Table A3—Appendix C). Summarizing the information contained in the selected articles, five robots are described.

Robot 1 is presented as a modular system, which is composed of sixteen aluminum modules made of laser-cut plastic with parallel joint axes, restricting the snake’s movement to only two dimensions. It is covered by multi-material skin that includes nylons, polyester, vinyl, mesh, and microfiber [81]. Also, there is a Robot 2 with fins and a drill (made of 3D-printed PLA) to excavate and move underground. The fins attached to the side of the body are anchored into the tunnel wall by rolling; meanwhile, the diameter of the drill bit is 45 mm relative to the width of the body 75 mm. The motors for the joints were Dynamixel XM430-W350R, while those for the drilling unit were XM540-W270R [82].

In addition, Robot 3, a soft-helix bodied system with more than 35 degrees of freedom at 35 cm in length, is actuated by a single rotary DC-motor to achieve the sidewinding, lateral undulation, accordion, linear movement gait [83]. Robot 4 performs multi-modal locomotion of side-winding (lateral undulation), concertina (the motors rotate in opposite directions to twist or untwist the spring), and side-pishing (the motors at both ends untwist the spring according to different rotation directions) using two rotary-motors [84]. There is also a two-degree-of-freedom system, Robot 5, with fiber-reinforced actuators connected in series. Each actuator has two chambers for bidirectional bending and an elliptical cross-section to prevent rolling. It is 20 cm in length, 3 cm in width, 2 cm in height and weighs 70 g [85,86].

After studying bio-inspired robotics based on “Snakes” that perform surface exploration, we select a particular species called Western Shovelnose Snake (*Sonora occipitalis*) [123], which is able to develop these steps for above-ground locomotion: it stretches out and anchors the front section of its body and then pulls up the rear, bunching itself into an ‘S’ pattern by propagating traveling waves down the body, head to tail [124]. It moves using a sideways swaying motion while it is either on or under the sand or loose soil [125]. 

#### 3.2.3. Spider

Starting with the search methodology (Table A2—Appendix B), in the identification stage, 62 matching articles were found in the Scopus database. Subsequently, in the screening stage, 58 articles were excluded as they did not meet the study analysis criteria (Table 2), leading to 4 articles (Figure 1 and Table A3—Appendix C). Summarizing the information presented in the selected articles, the robot designs present legged systems that perform rolling locomotion, which has the potential for increasing speed and agility compared to traditional wheeled rovers. Thus, two robots are described.

Robot 1 has a reconfigurable mechanism, which has four legs that are separated by an optimum distance from the body to ensure a smooth transformation from rolling and crawling [87]. The whole prototype is 3D manufactured using PLA material 3D printing [88]. Each limb has three servo motors that can deliver rotational motion on the pitch, yaw, and roll axis [89]. In addition, Robot 2 shows a 12-legged system driven by a pendulum combined with a flywheel, which served as the primary driving mechanism, while the flywheel was used for stability and as a means of supplying a propulsive forward force by releasing rotational kinetic energy. Additionally, simple radial legs powered by torsional springs were added to increase the effectiveness of long-distance travel over rugged terrain [90]. 

After studying bio-inspired robotics based on “Spiders” that perform surface exploration, we select a particular species called Golden Wheel Spider (*Carparachne aureoflava*) [126], which is able to develop these steps for above-ground locomotion; it reconfigures its body structures, like wheels taking the “tumbleweed” shape that ensures its ability to roll by fixing its legs into constant positions after a short runup and goes down sand-dunes. It resumes walking with its legs straight as rotational speed reduces [127].

## 4. Discussion

Bio-inspired robotics are prepared to revolutionize lunar exploration, particularly through designs inspired by terrestrial animals adapted to sand and soil environments [128]. These systems leverage advancements in mechanics and material science, and mechatronics and control systems to navigate and operate efficiently on the Moon’s surface and subsurface [129,130]. Such innovations promise to enhance our understanding of lunar geology, facilitate resource extraction, and support long-term human presence [131,132].

The exploration and utilization of space require advanced robotic systems capable of operating in harsh and unpredictable environments. Two prominent approaches in the design of space robots are “bio-inspired robots” and “conventional robots”. So, this section provides a comparison between these two types, examining their respective advantages and disadvantages (Table 4). By understanding the unique attributes of each, we can better determine their applicability in various space missions. The challenges of operating in the vacuum of space, dealing with extreme temperatures, and navigating uneven terrains necessitate sophisticated robotic designs. Bio-inspired robots, which draw design principles from nature, and conventional robots rely on traditional engineering techniques. Regarding robotics design and prototype integration, it is observed the potential of two fields:

(I) Mechanics and Materials Science: robots inspired by terrestrial animals adapted to sand and soil exhibit exceptional mechanical properties that facilitate efficient locomotion in granular media [133,134]. The incorporation of mechanical design using digital twins [135] related to compliant limbs and flexible spines [136], as seen in soft robotics, reduces ground reaction forces, improving stability and efficiency when navigating uneven terrains [137,138]. Burrowing robots that employ digging and peristaltic motions to move through sand [139], could achieve penetration depths in simulated lunar regolith [140], also innovative digging mechanisms inspired by origami-shaped bio-systems [141,142] can rotate the head modules with coupled soil-cutting blades [67], this behavior can improve energy efficiency compared to traditional drilling techniques, crucial for subsurface exploration [143,144].
  Moreover, modular [145,146] and reconfigurable [147,148] segments combined with sensors and actuators [149] provide redundancy and resilience against mechanical failures [150], ensuring continuous operation even under challenging conditions [151,152]. In addition, materials applications with the use of shape-memory alloys (SMAs) and other smart materials [153] enable robots to adapt their morphology to different tasks [154]. These can change their stiffness or shape in response to temperature variations, maintaining optimal performance under the Moon’s extreme conditions [155,156]. SMAs, for example, can recover pre-defined shapes with strain recovery, providing versatility in navigating tight spaces or overcoming obstacles [157,158]. Robots incorporating these soft materials [159] demonstrate increased adaptability and reduced mechanical failures [160,161]. Additionally, lightweight composite materials, such as carbon fiber-reinforced polymers [162], offer high strength-to-weight ratios, essential for minimizing launch costs and maximizing payload capacity [163]. These materials exhibit tensile strengths while maintaining low densities [164].
(II) Mechatronics and Control Systems: sensors [165] and actuators [166] designed to mimic the sensory and motor capabilities of terrestrial animals are crucial [167,168]. For example, some systems use micro-electromechanical systems (MEMS) [169,170] to replicate the adhesive properties on feet provided by adhesion forces [171]. Also, lightweight and high-torque actuators based on piezoelectric materials [172] provide precise and powerful movements [173,174], essential for both surface navigation and subsurface operations on Moon’s rugged topography [175]. In addition, artificial intelligence [176,177] and machine learning [178] define the autonomy and adaptability of bio-inspired robots [179,180]. Deep reinforcement algorithms [181,182] enable real-time decision-making and path planning [183], essential for navigating the unpredictable lunar terrain [184] and reducing energy consumption [185,186], while deep learning models [187,188] analyze sensor data to optimize locomotion and improve obstacle avoidance [189], enhancing overall mission success rates [190,191]. This capability enhances the efficiency and scope of lunar exploration missions, allowing for more comprehensive data collection and analysis [192]. In addition, convolutional neural networks (CNNs) can be employed for terrain classification of regolith types [193]. Additionally, AI-driven fault detection systems [194] enhance the reliability of robotic operations by predicting and mitigating failures before they occur [195]. Predictive maintenance algorithms have reduced downtime in terrestrial testing environments [196]. Furthermore, decentralized control systems [197], inspired by the collective behavior of insect colonies [198], allow for robust multi-robot cooperation (swarm systems) [199,200], facilitating large-scale exploration missions. These systems are being developed to improve task completion in simulated lunar environments [201,202].

**Table 4:** Space Planetary Robotics Comparison.

		Bio-Inspired Systems	Conventional Systems	
Mobility [150,203,204,205]	**ADVANTAGES**	They exhibit superior adaptability to varying and unpredictable environments. For instance, legged robots inspired by animals can traverse rough terrains more effectively than wheeled robots. It is associated with modularity, reconfigurability, and flexibility.	Those with wheels or tracks may struggle with uneven terrains and obstacles that bio-inspired robots can navigate more easily.	**DISADVANTAGES**
Energy [206,207,208,209]		They are focused on energy-efficient locomotion. For example, robots that emulate the movement of fish or birds use less energy for propulsion compared to conventional thrusters.	Conventional propulsion and locomotion methods often consume more energy, which is a critical concern in the energy-limited space environment.	
Size and Weight [35,210,211,212]		Mimicking the structure of small organisms can lead to the development of highly miniaturized robots. These micro-robots can perform tasks in tight spaces that larger robots cannot access.	They can be bulkier and heavier, making them less suitable for missions requiring compact and lightweight equipment.	
Development [213,214,215,216,217]	**DISADVANTAGES**	The intricate designs of bio-inspired robots often result in increased mechanical complexity. This can lead to higher production costs and more challenging maintenance.	They are often simpler in design and easier to manufacture. This simplicity can translate to lower costs and more reliable performance.	**ADVANTAGES**
Control Systems [218,219,220,221]		They replicate accurate biological behaviors that require advanced complex algorithms and significant computational power.	They are less complex and more intuitive, making them easier to operate and program.	
Proven Technology [222,223,224,225] and TRL [226,227]		There is a lack of mobile robots that have been created to be used on the Moon. However, some space robots have proven inherently robust and capable of self-repair to some extent. They often incorporate these traits, leading to more resilient and long-lasting systems.	The technologies used in these robots are well-established, reducing the risk of failures. This makes them suitable for critical missions where reliability is paramount.	

*Note: TRL (Technology Readiness Levels).*

**Figure 3:**
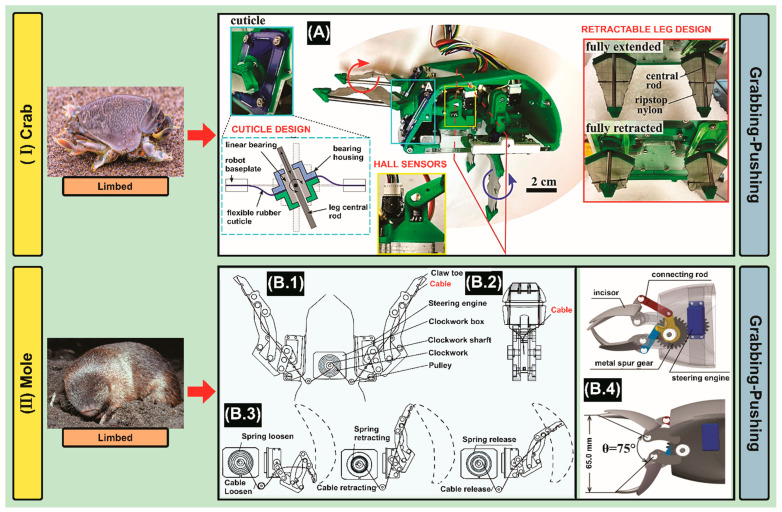
*Subsurface Exploration*: (**I**) Crab, Emerita Analoga (Standard Copyright Licence transferred to the authors) Adapted with permission from Bandersnatch(1808981506)/Shutterstock.com (accessed on 8 July 2024).—(**A**) Hardware components and full assembly, including the cuticle design, homing hall effect sensors, and retractable fabric leg design. Reproduced from [48]. CC BY 4.0. (**II**) Mole, Eremitalpa Granti (Standard Copyright Licence transferred to the authors) Adapted with permission from Anthony Bannister(MFFHY0)/Shutterstock.com (accessed on 9 July 2024).—(**B.1**) Design of the cable-driven burrowing force amplification mechanism. (**B.2**) System configuration. Reprinted from [50], Copyright (2023), with permission from IEEE. (**B.3**) Motion process during burrowing. (**B.4**) Prototype experiment and model angle measurement. Reprinted from [52], Copyright (2023), with permission from IEEE. Note: The left column shows the animal, while the right column represents the bio-inspired robot.

**Figure 4:**
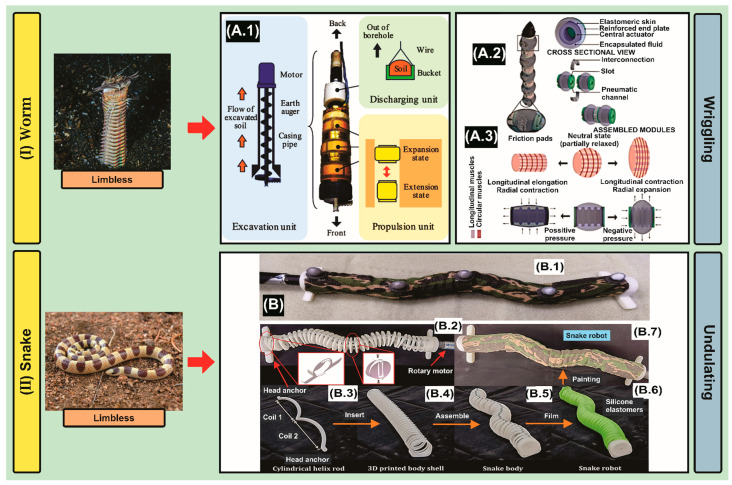
Subsurface Exploration: (**I**) Worm, Eunice Aphroditois (Standard Copyright License transferred to the authors) Adapted with permission from Cingular(1219459138)/Shutterstock.com (accessed on 8 July 2024).—(**A.1**) Robot is mainly made up of three units: a propulsion unit, an excavation unit, and a discharging unit. The propulsion unit contains three additional propulsion subunits and propels through a borehole by reproducing the peristaltic crawling motion of an earthworm. Moreover, the propulsion unit allows LEAVO to excavate deep underground by supporting the reaction torque/force of the excavation by gripping the wall of the borehole. The excavation unit mainly includes an excavation instrument, namely, an “earth auger”, and a casing pipe covering the earth auger. The excavation unit excavates soil and transports it to the back of the robot. The soil in the back of the robot is discharged out of the borehole using the discharging unit. Reprinted from [59], Copyright (2018), with permission from IEEE. (**A.2**) Bio-inspired PSA modules are assembled in series using interconnections to form a soft robot with passive setae-like friction pads on its ventral side. (**A.3**) Working principle of the actuator with positive and negative pressure compared to the muscular motion observed in earthworm segments. Reproduced from [69]. CC BY 4.0. Surface Exploration: (**II**) Snake, Sonora Occipitalis (Standard Copyright License transferred to the authors) Adapted with permission from Matt Jeppson(86483413)/Shutterstock.com (accessed on 8 July 2024).—(**B.1**) An overview of the snake robot locomotion experiment. The snake robot is moving on granular terrain. A single DC motor drives the robot to generate sidewinding locomotion. The motion capture system captures the motion data through five reflective markers on the snake robot. (**B.2**) Fabrication of the continuous snake robot with a single rotary motor. Different mounting holes on the head anchor are used to adjust the slope angle. Basins assemble the body shells. (**B.3**) A cylindrical helix rod with two coils is made by 3D printing. (**B.4**) 3D printed body shells are linked to form a robot snake shell. (**B.5**) the helix rod is put into the body shells to form the snake robot body. (**B.6**) The snake robot body is filmed with silicone elastomers to improve the friction coefficient; (**B.7**) Prototype of snake robot after painting. Reprinted from [83], Copyright (2023), with permission from IEEE. Note: The left column shows the animal, while the right column represents the bio-inspired robot.

**Figure 5:**
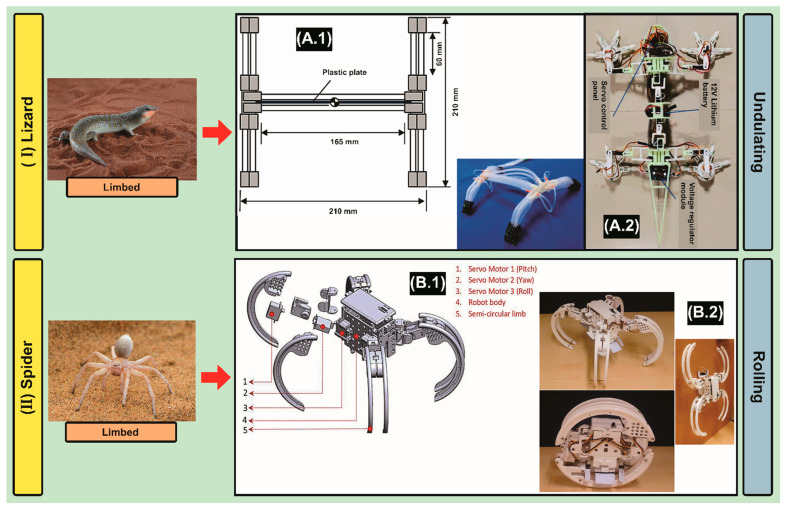
Surface Exploration: (**I**) Lizard, Scincus Scincus (Standard Copyright License transferred to the authors) Adapted with permission from Kurit afshen(2358731213)/Shutterstock.com, (accessed on 8 July 2024).—(**A.1**) schematic of robot design—top view, and soft-amphibious robot-Reprinted from [77], Copyright (2017), with permission from IEEE. (**A.2**) fabricated prototype of the lizard-inspired quadruped robot moving on simulated Mars surface terrains. Reproduced from [79]. CC BY 4.0. (**II**) Spider, Carparachne Aureoflava (Standard Copyright License transferred to the authors) Adapted with permission from Tobias Hauke(1958871052)/Shutterstock.com (accessed on 8 July 2024).—(**B.1**) 4 legged-system showing the pitch, roll, and yaw servo motors associated with the hemispherical limbs while the robot is in the crawling posture. (**B.2**) Bio-inspired reconfigurable prototype. Reproduced from [88]. CC BY 4.0. Note: The left column shows the animal, while the right column represents the bio-inspired robot.

Finally, according to the aforementioned information described in this section, we can state a bio-robot design proposal that covers the minimum optimized requirements related to the morphology of the animal species replicated in the mechatronic systems presented in Section 3. In summary, our robot would have the following characteristics based on the type of exploration in sandy substrates: (1) Subsurface: the front legs of the crab to initiate submersion (Figure 3(IA)), and then, the mole’s arms positioned in parallel to dig and clear the path (Figure 3(IIB.1)); in addition, the main body that mimics the worm anatomy (which serves for sample collection, and then analyzes it in-situ using biosensors [228,229]) will contain the excavation and propulsion unit (Figure 4(IA.1)); (2) Surface: the fastest option for locomotion is the wheel-type system, so the best option is the geometry of the spider leg’s structure (Figure 5(IIB.2)). Finally, we can conclude that our robot will be capable of grabbing, pushing, and wriggling for subsurface exploration, followed by rolling for surface exploration.

## 5. Conclusions

There is no known bio-inspired robot that currently works on the Moon that has been published at any conference, journal, or book; however, there are very interesting developments related to animal morphology biomimicry that have been tested in analog environments that replicate some planetary conditions, such as regolith simulant. Biomimetics and bio-inspiration improve products and procedures across a wide range of industries. Thus, several features shared by biological organisms, such as autonomy and intelligence, response-stimuli adaptability, energy efficiency, ability to self-repair, robustness, lightweight construction, and digging tasks, are essential for space systems. Therefore, the highlights of this study cover: (I) A scoping review oriented to the design of animal bio-inspired robotic hardware that could be used for planetary exploration (Until July 2024). It was based on bibliometric analysis, where papers were reviewed from Scopus. Thus, a total of 482 studies were selected. (II) Classification and comparison of limbed and limbless systems for subsurface or surface exploration derived from the morphology of soil/sand-adapted animals, where the most published types of robots are based on the knowledge from worms (7), moles (6), snakes (5), lizards (4), crabs (2), and spiders (2). The most published articles were included in the International Conference on Advanced Intelligent Mechatronics (AIM) by IEEE/ASME Publisher, and Bioinspiration & Biomimetics Journal by IOP Science Publisher. (III) Propose a pioneering methodology to design bio-inspired robots, justifying the application of biological principles for lunar exploration, due to technical features related to actuators, sensors, and mechanisms shown in Figure 3, Figure 4 and Figure 5. Finally, space robotics are constantly growing with findings that can inspire new methods to design animal-morphing biomechatronic systems.

## Figures and Tables

**Table 1:** Summary of PICOC.

**Population**	The literature on biorobotic animal-shape morphologies that improve grabbing-pushing, undulating, rolling, or wriggling tasks on the sand.
**Intervention**	Biomimetic systems with semi-autonomous or autonomous animal-like locomotion.
**Comparison**	Limbed and limbless animal species
**Outcomes**	Adaptive morphologies considering subsurface and surface locomotion
**Context**	Studies in industry and academia, small and large data sets

**Table 2:** Eligibility Selection Criteria of Published Papers.

**Inclusion** **Criteria**	Full text of the article is available
	The publication year is up to July 2024
	Article is about a mobile robot with an animal’s shape and morphology
	Article is about hardware design
	Article is about a manufacturing/prototyping process
	Article is about robot animal’s locomotion
	Article is written in English
**Exclusion** **Criteria**	Article is a review paper

**Table 3:** Selected Sand-Adapted Animals Replicated by Biomimetic Robots.

Exploration /Locomotion		Animal (Species)	Scientific Name	Robot #	Authors	Citation	YP		
**SUBSURFACE**	**GRABBING-PUSHING**	CRAB (Pacific Mole Crab)	*Emerita analoga*	1	L. K. Treers et al.	[48]	2022	2	**BIO-INSPIRED MECHATRONIC SYSTEMS**
				2	R. A. Russell et al.	[49]	2011		
		MOLE (Grant’s Golden Mole)	*Eremitalpa granti*	1	Z. Liang et al.	[50]	2023	6	
				2	J. Lee, J. Kim et al.	[51]	2020		
				3	H. Zheng et al.	[52]	2023		
				4	J. Lee et al.	[53,54,55,56]	2019, 2020, 2022		
				5	C. Tirtawardhana et al.	[57]	2020		
				6	J. Kim et al.	[58]	2018		
	**WRIGGLING**	WORM (Bobbit worm)	*Eunice aphroditois*	1	T. Nakamura et al.	[59,60,61,62,63,64]	2009, 2017–2019, 2021, 2022	7	
				2	P. Zhang et al.	[65]	2024		
				3	B. Liu et al.	[66]	2019		
				4	H. Fang et al.	[67]	2017		
				5	A. A. Calderón et al.	[68]	2019		
				6	R. Das et al.	[69,70]	2023		
				7	Y. Ozkan-Aydin et al.	[71]	2021		
**SURFACE**	**UNDULATING**	LIZARD (Sandfish Lizard)	*Scincus scincus*	1	R. D. Maladen et al.; and J. Urquhart	[72,73,74,75,76]	2010, 2011	4	
				2	A. A. M. Faudzi et al.	[77]	2017		
				3	B. Chong et al.	[78]	2022		
				4	G. Chen et al.	[79,80]	2024		
		SNAKE (Western Shovelnose Snake)	*Sonora occipitalis*	1	C. Wright et al.	[81]	2007	5	
				2	H. Yoshida et al.	[82]	2023		
				3	L. Huang et al.	[83]	2023		
				4	W. Zhao et al.	[84]	2021		
				5	C. Branyan et al.	[85,86]	2017, 2018		
	**ROLLING**	SPIDER (Golden Wheel Spider)	*Carparachne aureoflava*	1	R. Elara Mohan et al.	[87,88,89]	2015–2017	2	
				2	A. Western et al.	[90]	2023		

## Data Availability

The original contributions presented in the study are included in the article. Further inquiries can be directed to the corresponding authors.

## Abbreviations

The following abbreviations are used in this manuscript:

ABS	Acrylonitrile Butadiene Styrene
CNNs	Convolutional Neural Networks
ER	Extraterrestrial Regolith
ISRU	In-situ Resource Utilization
MEMS	Micro-Electromechanical Systems
NASA	National Aeronautics and Space Administration
PETE	Polyethylene terephthalate
PICOC	Population, Intervention, Comparison, Outcomes, and Context
PLA	Polylactic Acid
PRIME-1	Polar Resources Ice Mining Experiment
PRS	Planetary Regolith Sampler
PSA	Peristaltic-Soft Actuator
SMAs	Shape-Memory Alloys
SF-X	Surface Exploration
SSF-X	Subsurface Exploration
TRL	Technology Readiness Levels
VIPER	Volatiles Investigating Polar Exploration Rover
YP	Year of Publication

## Appendix A

**Table A1:** Search Protocol.

Structure		Keyword–Queries/Search String	
**BLOCK 1**	**PART 1**	(Common Name)	TITLE-ABS
		OR	
		(Scientific name)	
		(Family name)	
	AND		
	**PART 2**	(“robot”)	
AND			
**BLOCK 2**	**PART 3**	(“Excavation” OR “Digging” OR “Drilling” OR “Burrowing”)	ABS
	OR		
	**PART 4**	(“Sand” OR “Subsurface” OR “Underground” OR “Subterranean” OR “Subterrestrial”)	ABS
	OR		
	**PART 5**	(“Terrain” or “Sand”)	ABS

## Appendix B

**Table A2:** Search string used in Scopus for each animal.

	Key-Words	Animal
TITLE-ABS	((crab OR “emerita analoga” OR “hippidae”) AND robot) AND (ABS (excavation OR digging OR drilling OR burrowing) OR ABS (subsurface OR underground OR subterranean OR subterrestrial) OR ABS (terrain OR sand))	CRAB
	((mole OR “eremitalpa granti” OR “ chrysochloridae”) AND robot) AND (ABS (excavation OR digging OR drilling OR burrowing) OR ABS (subsurface OR underground OR subterranean OR subterrestrial) OR ABS (terrain OR sand))	MOLE
	(((earthworm OR “lumbrineris latreilli” OR “lumbrineridae”) OR (“bobbit worm” OR “Eunice aphroditois”)) AND robot) AND (ABS (excavation OR digging OR drilling OR burrowing) OR ABS (subsurface OR underground OR subterranean OR subterrestrial) OR ABS (terrain OR sand)))	WORM
	((lizard OR “scincus scincus” OR “scincidae”) AND robot) AND (ABS (excavation OR digging OR drilling OR burrowing) OR ABS (subsurface OR underground OR subterranean OR subterrestrial) OR ABS (terrain OR sand))	LIZARD
	((snake OR “sonora occipitalis” OR “colubridae”) AND robot) AND (ABS (excavation OR digging OR drilling OR burrowing) OR ABS (subsurface OR underground OR subterranean OR subterrestrial) OR ABS (terrain OR sand))	SNAKE
	((spider OR “Carparachne aureoflava” OR “sparassidae”) AND robot) AND (ABS (excavation OR digging OR drilling OR burrowing) OR ABS (subsurface OR underground OR subterranean OR subterrestrial) OR ABS (terrain OR sand))	SPIDER

## Appendix C

**Table A3:** List of conferences, journals, and book publications regarding selected articles of biorobotics that could be used for subsurface and surface exploration on the Moon. (#: number of papers).

		Animal	PUBLICATION MEDIA	TYPE	PUBLISHER	#
**SOIL/SAND ADAPTED**	**SUBSURFACE EXPLORATION**	CRAB	Frontiers in Robotics and AI	Journal	Frontiers	1
			Advanced Robotics	Journal	Taylor & Francis	1
		MOLE	International Conference on Advanced Robotics and Mechatronics (ICARM)	Conference	IEEE	1
			International Conference on Robot Intelligence Technology and Applications (RiTA)	Conference	Springer	4
			International Conference on Mechatronics and Automation (ICMA)	Conference	IEEE	1
			International Conference on Control, Automation and Systems (ICCAS)	Conference	IEEE	1
			International Conference on Intelligent Robots and Systems (IROS)	Conference	IEEE	1
			IEEE Access	Journal	IEEE	1
		WORM	International Conference on Advanced Intelligent Mechatronics (AIM)	Conference	IEEE/ASME	3
			International Conference on Soft Robotics (RoboSoft)	Conference	IEEE	2
			Active and passive smart structures and integrated systems	Conference	SPIE	1
			Climbing and Walking Robots Conference (CLAWAR)	Conference	Springer	1
			IEEE Access	Journal	IEEE	1
			Industrial Robot: An International Journal	Journal	Emerald	1
			Marine Georesources & Geotechnology	Journal	Taylor & Francis	1
			Bioinspiration & biomimetics	Journal	IOP Science	2
			Scientific Reports	Journal	Nature	1
	**SURFACE EXPLORATION**	LIZARD	International Conference on Robotics and Automation	Conference	IEEE	1
			International Conference on Advanced Intelligent Mechatronics (AIM)	Conference	IEEE/ASME	1
			Proceedings of the National Academy of Sciences	Conference	NAS	1
			MIT Press Direct	Book Chapter	MIT	1
			The International Journal of Robotics Research	Journal	SAGE	1
			New Scientist	Journal	DMGT	1
			Journal of The Royal Society Interface	Journal	Royal Society	1
			Bioinspiration & Biomimetics	Journal	IOP Science	2
		SNAKE	International Conference on Intelligent Robots and Systems,	Conference	IEEE	2
			International Conference on Advanced Intelligent Mechatronics (AIM)	Conference	IEEE/ASME	1
			International Conference on Robotics and Biomimetics (ROBIO)	Conference	IEEE	1
			IEEE/ASME Transactions on Mechatronics	Journal	IEEE/ASME	1
			Advanced Robotics	Journal	Taylor & Francis	1
		SPIDER	International Conference on Engineering Design (ICED)	Conference	The Design Society	1
			International Journal of Advanced Robotic Systems	Journal	SAGE	1
			Applied Sciences	Journal	MDPI	1
			Acta Astronautica	Journal	Elsevier	1
